# Draft genome of pathogenic heterotrophic bacterium *Bacillus altitudinis* 2R-9

**DOI:** 10.1128/mra.00436-23

**Published:** 2024-01-10

**Authors:** Lesego G. Molale-Tom, Rinaldo K. Kritzinger, Oluwaseyi S. Olanrewaju, Cornelius C. Bezuidenhout

**Affiliations:** 1 Unit for Environmental Sciences and Management, Potchefstroom Campus, North-West University, Potchefstroom, North-West, South Africa; DOE Joint Genome Institute, Berkeley, California, USA

**Keywords:** *Bacillus*, genome analysis, water treatment plants, South Africa

## Abstract

Human activity affects the quality of potable water sources and their associated bacterial communities. Here, we discuss the heterotrophic *Bacillus altitudinis* 2R-9 draft isolated from the raw source of a drinking water distribution system in South Africa.

## ANNOUNCEMENT

*Bacillus altitudinis* is a bacterial species isolated from various sources, including honey, bee bread, propolis, plant roots, and fish intestines (1). *B. altitudinis* has potential biotechnological applications, such as enhancing the accumulation of ginsenosides in *Panax ginseng* (2) and inhibiting the growth of *Alternaria alternata in vitro* (3). It has also been discovered to be beneficial for *P. ginseng* growth and morbidity reduction (4). Furthermore, it has been demonstrated that *B. altitudinis* can produce thermostable β−1,3–1,4-glucanase (5), and its genome has been sequenced (6). Lastly, research on antimicrobial resistance has illustrated *B. altitudinis* could be resistant to some antibiotics (7).

The *B. altitudinis* strain reported was isolated from the untreated water source for a drinking water distribution plant in the North-West province of South Africa in August 2016. The strain was inoculated on nutrient agar and incubated at 37℃ for 24 hours. The genomic DNA was extracted using the Chemagic DNA Bacteria Kit (PerkinElmer, Germany) following the manufacturer’s protocol. DNA concentration was quantified using a Nanodrop 1000 spectrophotometer (Thermo Fisher Scientific, USA), and the library was performed using the Nextera XT kit (Illumina Inc., San Diego, CA) according to the instructions provided by the manufacturer and sequenced using Illumina’s MiSeq300 in paired-end reads at the North-West University sequencing facility. The generated raw paired-end fastq reads (2 × 300 bp) were quality checked using FastQC v.0.11.7 (8) followed by trimming of low-quality bases using Trimmomatic v.0.39 (9), and the data quality was rechecked using FastQC v.0.11.7 (8). The cleaned reads were assembled using SPAdes v.3.15.5 (10). Quast (v.5.0.2) (11) was used to evaluate the genome assembly quality. The completeness and contamination were assessed with CheckM (v.1.1.6) (12). The assembled draft genome was annotated on the Rapid Annotation System Technology (RAST) Pipeline (13) and NCBI Prokaryotic Genome Annotation Pipeline (PGAP) v6.5(14). The annotated genomes were assessed against the Genome Taxonomy Database (GTDB) using GTDB-Tk software v1.7.0 (15). Default parameters were used for all software except where otherwise noted.

**Fig 1:**
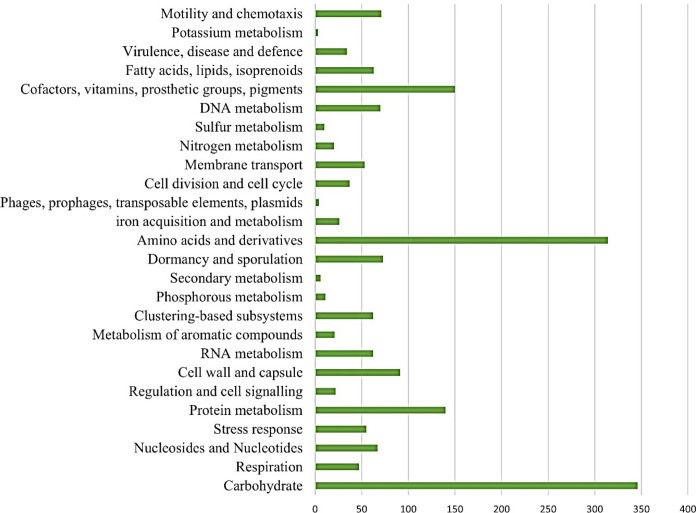
Functional annotation categories in the genome based on RAST annotation.

A total of 2,356,906 read counts and coverage of 130.7× were generated. The total length of the draft genome was 3,762,390 bp; it included 81 contigs, 41.1% GC content, N50 and L50 of 375,146 and 3, respectively. According to CheckM, the genome is 99.59% complete. The annotation revealed various functional genes showing important functions, including motility, virulence, and presence of mobile genetic elements such as phages, prophages, plasmids, and transposable elements (Fig. 1). The prediction of gene functions based on PGAP annotation revealed 3,897 genes, including 3,772 protein-coding genes and 3,795 coding DNA sequences (CDSs), 102 total rRNAs, and 74 tRNAs.

In conclusion, the pathogenicity of this bacterial species on humans has not been established; therefore, this new genome will contribute further insight into the biology and diversity of this heterotrophic bacterial species.

## Data Availability

The whole-genome shotgun project for *Bacillus altitudinis* 2R-9 has been deposited at DDBJ/ENA/GenBank under the accession JASCXE000000000, and the version described in this paper is version JASCXE010000000. The raw reads are available under the BioProject accession number PRJNA968034, and the BioSample accession number is SAMN34894825. The sequence data obtained in this work have been deposited in the NCBI Sequence Read Archive under the accession number SRR24490156.
